# A Phase-Coded Sequence Design Method for Active Sonar

**DOI:** 10.3390/s20174659

**Published:** 2020-08-19

**Authors:** Chengyu Guan, Zemin Zhou, Xinwu Zeng

**Affiliations:** College of Meteorology and Oceanology, National University of Defense Technology Changsha, Changsha 410000, China; guan_nudt@nudt.edu.cn (C.G.); zzm@nudt.edu.cn (Z.Z.)

**Keywords:** active sonar, periodic autocorrelation, weighted integrated sidelobe level, majorization–minimization, sequence design

## Abstract

Phase-coded sequences are widely studied as the transmitted signals of active sonars. Recently, several design methods have been developed to generate phased-coded sequences satisfying specific aperiodic or periodic autocorrelation sidelobe level metrics. In this paper, based on the majorization–minimization strategy and the squared iterative acceleration scheme, we propose a method to generate sequences with the periodic weighted integrated sidelobe level metric. Numerical simulations illustrate that the proposed method can effectively suppress the periodic autocorrelation sidelobe levels in specific time lags. Compared with other sequence design methods satisfying the periodic weighted integrated sidelobe level metric, our method improves the computational efficiency significantly. In addition, the proposed sequence demonstrates better matched filter performance in specific range intervals compared with its counterpart. The results suggest that the method could be applied as a valid and real-time design method for transmitted signals of active sonars.

## 1. Introduction

The autocorrelations of transmitted signals are of great significance for the signal processing in active sonars. A good autocorrelation property indicates that the signal is nearly uncorrelated with its own time-delay versions [1,2], which ensures that the active sonar can precisely extract the echo information from the interested time lags while suppressing interferences from other time lags [3,4]. One of the most widely used transmitted signals with good autocorrelation properties is the phase-coded sequence [5,6]. Due to practical constraints of sonar transducers such as the frequency response and the energy efficiency, it is desirable to design the transmitted signals with nearly a constant amplitude, which aroused a lot of effort to research on unimodular phase-coded sequences [7,8]. The early studies regarding phase-coded sequences mainly focused on binary sequences to reach the low autocorrelation sidelobes, for example, the Barker sequence [9]. Lately, considering the fact that binary sequences are of low computational efficiency and difficult to be generated with long length, researches shifted to other polyphase sequences such as the Golomb sequences [10,11] and the Frank sequences [12]. Correspondingly, the optimization for polyphase sequences becomes one of the major interests in the sequence designs. Most researchers optimize this problem with the Integrated Sidelobe Level (ISL) metric [13,14,15] or the Weighted Integrated Sidelobe Level (WISL) metric [11,16,17].

The ISL metric is the most commonly-used criterion to evaluate the autocorrelation properties of phase-coded sequences. Based on the singular value decomposition (SVD), Li first proposed the cyclic algorithm (CA) to design sequences satisfying the ISL metric [18]. However, as the SVD operations are relatively computationally intensive, it might be difficult to generate the sequence with length more than $N^{3}$. Afterwards, Stoica developed two extensions of the CA, called ‘Cyclic Algorithm New’ (CAN) and ‘Periodic-correlation Cyclic Algorithm New’ (PeCAN) to generate sequences of length $N\sim 10^{6}$ in the aperiodic and periodic ISL case, respectively [13,14]. Compared with the CA, the CAN and the PeCAN utilize the Fast Fourier Transform (FFT) operations and reduce computational burdens effectively. 

For a phase-coded sequence satisfying the ISL metric, it is difficult and time-consuming to suppress autocorrelation sidelobes at all time lags. As a result, the WISL metric is developed to reduce autocorrelation sidelobe levels at specific time lags [13]. Hao He proposed the periodic CA (PeCA) to generate sequence sets with the periodic WISL metric based on the SVD operations [19]. Stoica developed an algorithm called ‘Weighted Cyclic Algorithm New’ (WeCAN) for the design of sequences with the aperiodic WISL metric [13]. Compared with sequences satisfying the ISL metric, the PeCA and the WeCAN sequences are flexible in practical applications.

Although the above algorithms, PeCA and WeCAN, can generate sequences with the WISL metric, the computational burdens are considerably intensive. In order to enhance the computational efficiency, the majorization–minimization (MM) strategy was applied to sequence design methods. Instead of optimizing the objective problem directly, the MM strategy constructs surrogate Equations to approximate the objective and gradually decomposes the N-dimensional equation into the sum of one-dimensional equations which can be minimized easily [20]. This strategy has already been implemented to large-scale or non-convex optimization problems [21]. Song dealt with the aperiodic ISL and WISL Equations through the MM strategy [15,17]. Compared with the aforementioned CA and CAN, the algorithm using MM strategy reaches a faster convergence speed and lower computational burden.

In this paper, we intended to apply the MM strategy for the periodic WISL Equation and proposed a real-time sequence design method. The main contributions and advantages of the paper can be summarized as: (1) We intended to reach low autocorrelation sidelobes at specific time lags (namely WISL metric), which is much easier to reach than the ISL metric in the complex underwater environment we focus on. (2) In this paper, we reduced the calculation amount and improved the real-time capacity of sequence design methods in periodic WISL case, since the recent methods generating sequences with the periodic WISL cannot be satisfactory [19]. (3) For the first time, we proved that the periodic WISL Equation can be tackled through the MM strategy which has been applied in the aperiodic/periodic ISL and the aperiodic WISL equation as for example in [15,16,17].

The rest of the paper is organized as follows. In Section 2, we derived a phased-coded sequence design method based on the MM strategy. Some derivations of our method in periodic WISL case are different from the aperiodic case [17]. For completeness and clearness, we presented all derivations of the method in this section. Also, an acceleration scheme was applied in the method in order to enhance the convergence speed. Simulations are presented in Section 3 to evaluate the convergence performance of the proposed method. In addition, the matched filter performance of the transmitted sequence was evaluated since the matched filter is a standard echo processing of active sonars. Finally, Section 4 proposed the conclusions of this paper.

Notation: In this paper, boldface upper case letters denote matrices, boldface lower case letters denote column vectors. Notations ${\left(\cdot \right)}^{T},{\left(\cdot \right)}^{*},{\left(\cdot \right)}^{H}$ denote transpose, conjugate, and conjugate transpose. Re(·) denotes the real part, Tr(·) denotes the trace of the matrix and vec(·) denotes the column-wise vectorization. ${\left\|\cdot \right\|}_{2}$ denotes the Euclidean norm. Diag(x) is a diagonal matrix formed with x as its principal diagonal. I is the identity matrix.

## 2. MM Based Phase-Coded Sequence Design Method 

Let’s define $x=\left\{x_{n}|n=1,\ldots N\right\}$ as a complex unimodular sequence with length N. It is well known that the periodic autocorrelation of the sequence can be defined as [13]:

(1) $$r_{k}=\sum_{n=1}^{N}x_{n}x_{\left(n-k\right)\mathrm{mod}N}^{*}=r_{-k}^{*},k=0,\ldots ,N-1$$

Here nmodN is the modulo operation which denotes that:

(2) nmodN=n−⌊n/N⌋N

where ⌊n/N⌋ is the largest integer smaller than or equal to n/N. The WISL metric of periodic autocorrelation can be written as:

(3) $$\mathrm{WISL}=\sum_{k=1}^{N-1}\omega_{k}{\left|r_{k}\right|}^{2}$$

where $\omega_{k},k=1,\ldots ,N-1$ represent the weights set of the WISL metric. Then the design method of the sequence x is considered as the following optimization problem:

(4) $$\begin{array}{ll} \underset{x_{n}}{\mathrm{minimize}} & \;\mathrm{WISL}\\ \mathrm{subject}\;\mathrm{to} & \;\left|x_{n}\right|=1,n=1,\ldots ,N \end{array}$$

Equation (4) expresses the optimization problem of both the aperiodic [17] and the periodic WISL metric. To make the WISL metric expressed clearly, the basis matrices in the aperiodic case are defined as N×N Toeplitz matrices $U_{k},k=0,\ldots ,N-1$:

(5) $$U_{k}=\left[\begin{array}{cccccc} u_{0} & u_{1} & \cdots & u_{k} & \cdots & u_{N-1}\\ u_{-1} & u_{0} & u_{1} & & & \vdots \\ \vdots & u_{-1} & \ddots & \ddots & & u_{k}\\ u_{-k} & & \ddots & u_{0} & u_{1} & \vdots \\ \vdots & & & u_{-1} & \ddots & u_{1}\\ u_{-\left(N-1\right)} & \cdots & u_{-k} & \cdots & u_{-1} & u_{0} \end{array}\right]$$

where $u_{k}$ in *k*th diagonal are 1 and elsewhere are 0 [17]. 

On the other hand, in periodic case, we define new basis matrices $V_{k},k=1,\ldots ,N-1$ as N×N circulant matrices:

(6) $$V_{k}=\left[\begin{array}{cccccc} v_{0} & v_{1} & \cdots & v_{k} & \cdots & v_{N-1}\\ v_{-\left(N-1\right)} & v_{0} & v_{1} & & & \vdots \\ \vdots & v_{-\left(N-1\right)} & \ddots & \ddots & & v_{k}\\ v_{-\left(N-k\right)} & & \ddots & v_{0} & v_{1} & \vdots \\ \vdots & & & v_{-\left(N-1\right)} & \ddots & v_{1}\\ v_{-1} & \cdots & v_{-\left(N-k\right)} & \cdots & v_{-\left(N-1\right)} & v_{0} \end{array}\right]$$

where $v_{k}$ in *k*th diagonal and $v_{-\left(N-k\right)}$ in −(*N − k*)th diagonal are all 1 and elsewhere are 0. To express Equation (4) as the symmetric form, let $V_{0}$ be a null matrix and further define $V_{-k}=V_{k}^{T},k=1-N,\ldots 0\ldots ,N-1$. Through the expression of basis matrices $V_{k}$, Equation (4) with WISL metric of (3) can be rewritten as:

(7) $$\begin{array}{ll} \underset{X,x}{\mathrm{minimize}} & \;\frac{1}{2}\sum_{k=1-N}^{N-1}\omega_{k}{\left|\mathrm{Tr}\left(V_{k}X\right)\right|}^{2}\\ \mathrm{subject}\;\mathrm{to} & \;X=xx^{H}\\ & \left|x_{n}\right|=1,n=1,\ldots ,N \end{array}$$

where $\omega_{-k}=\omega_{k},\omega_{0}=0$. Since $\mathrm{Tr}\left(V_{k}X\right)=\mathrm{vec}\left(V_{k}\right)\mathrm{vec}{\left(X\right)}^{H}$, Equation (7) can be expressed as:

(8) $$\underset{X,x}{\mathrm{minimize}}\;\sum_{k=1-N}^{N-1}\omega_{k}\mathrm{vec}{\left(X\right)}^{H}\mathrm{vec}\left(V_{k}\right)\mathrm{vec}{\left(V_{k}\right)}^{H}\mathrm{vec}\left(X\right)$$

Let’s define:

(9) $$R=\sum_{k=1-N}^{N-1}\omega_{k}\mathrm{vec}\left(V_{k}\right)\mathrm{vec}{\left(V_{k}\right)}^{H}$$

It is obvious that R is a Hermitian matrix. By the definition of R, Equation (8) can be rewritten as:

(10) $$\underset{X,x}{\mathrm{minimize}}\;\mathrm{vec}{\left(X\right)}^{H}R\mathrm{vec}\left(X\right)$$

In the following parts, Equation (10) will be tackled by using the MM strategy. The key procedures of the MM strategy include: (1) Majorization: constructing majorization functions u(M) as accurate as possible by designing an upperbound matrix M of the object matrix R. (2) Minimization: surrogating the original Equation (10) with the majorization function u(M) and minimizing the surrogate equation. These two procedures will repeat several times until the closed-form solution can be reached. Before we use the MM strategy, a useful conclusion [15] should be displayed so that it can be used later.

**Lemma** **1.** $R,M_{1}$*are both*N×N*Hermitian matrices and*$M_{1}\geq R$*. For each*$x_{0}\in {\mathbb{C}}^{N}$*, the majorization function of*$x^{H}Rx$ is xHM1x+2Re(xH(R−M1)x0)+x0H(M1−R)x0, *which ensures that* $x^{H}Rx$ $\leq x^{H}Mx$ +2Re(xH(R−M)x0) $+x_{0}^{H}\left(M-R\right)x_{0}$.

According to Lemma 1, an upperbound matrix $M_{1}$ which satisfying $M_{1}\geq R$ needs to be designed in order to construct the majorization function. A simple choice is to define $M_{1}=\lambda_{\max}\left(R\right)I$ where $\lambda_{\max}\left(R\right)$ is the maximum eigenvalue of R. The value of $\lambda_{\max}\left(R\right)$ is quantitatively expressed as:

(11) $$\lambda_{\max}\left(R\right)=\underset{k}{\max}\{N\left(\omega_{k}+\omega_{N-k}\right)k=1,\ldots ,N-1\}$$

**Proof.**  Owing to the property of circulant matrices, $\mathrm{vec}\left(V_{k}\right),k=1,\ldots ,N-1$ are mutually orthogonal so that:

(12) $$\mathrm{vec}{\left(V_{i}\right)}^{H}\mathrm{vec}\left(V_{j}\right)=\left\{ \begin{array}{l} \begin{array}{cc} 1, & \mathrm{when}\;i=j \end{array}\\ \begin{array}{cc} 0, & \mathrm{when}\;i\neq j \end{array} \end{array}\right.$$

Then both sides of Equation (9) are multiplied by $\mathrm{vec}\left(V_{k}\right)$:

(13) $$\begin{array}{lll} R\mathrm{vec}\left(V_{k}\right) & = & \sum_{j=1-N}^{N-1}\omega_{j}\mathrm{vec}\left(V_{j}\right)\mathrm{vec}{\left(V_{j}\right)}^{H}\mathrm{vec}\left(V_{k}\right)\\ & = & \omega_{k}\mathrm{vec}\left(V_{k}\right)\mathrm{vec}{\left(V_{k}\right)}^{H}\mathrm{vec}\left(V_{k}\right)+\\ & & \omega_{-\left(N-k\right)}\mathrm{vec}\left(V_{-\left(N-k\right)}\right)\mathrm{vec}{\left(V_{-\left(N-k\right)}\right)}^{H}\mathrm{vec}\left(V_{k}\right)\\ & = & N\left(\omega_{k}+\omega_{N-k}\right)\mathrm{vec}\left(V_{k}\right) \end{array}$$

where the second equality results from the fact $V_{k}=V_{-\left(N-k\right)}$ and the third equality results from the fact $\omega_{-\left(N-k\right)}=\omega_{N-k}$. According to Equation (13), $N\left(\omega_{k}+\omega_{N-k}\right)$ are non-zero eigenvalues of R with corresponding eigenvectors $\mathrm{vec}\left(V_{k}\right)$. Then the maximum eigenvalue of R is given as Equation (11).

Giving $X^{\left(p\right)}=x^{\left(p\right)}{\left(x^{\left(p\right)}\right)}^{H}$ of the *p*th iteration and choosing $M_{1}=\lambda_{\max}\left(R\right)I$. It is easy to see that both $M_{1}$ and R are Hermitian matrices. According to Lemma 1, the majorization function which surrogates Equation (10) at $X^{\left(p\right)}$ can be given as:

(14) u1(X,X(p))=λmax(R)vec(X)Hvec(X)+2Re(vec(X)H(R−M1)vec(X(p)))+vec(X(p))H(M1−R)vec(X(p))

Considering the fact that $\mathrm{vec}{\left(X\right)}^{H}\mathrm{vec}\left(X\right)={\left(x^{H}x\right)}^{2}=N^{2}$, the first term of Equation (14) is a constant and the third term depends on the *p*th iteration only. Ignoring these immaterial terms, Equation (10) can be surrogated by $u_{1}\left(X,X^{\left(p\right)}\right)$ as:

(15) minimizeX,x Re(vec(X)H(R−M1)vec(X(p)))

Substituting Equation (9) into Equation (15), the equation becomes:

(16) minimizeX,x ∑k=1−NN−1ωkRe(Tr(VkX)Tr(VkX(p))∗)−λmax(R)Tr(X(p)X)

Considering $\mathrm{Tr}{\left(V_{k}X^{\left(p\right)}\right)}^{*}={\left(r_{k}^{*}\right)}^{\left(p\right)}=r_{-k}^{\left(p\right)}$ and $X^{\left(p\right)}={\left(x^{\left(p\right)}\right)}^{H}x^{\left(p\right)}$, then we rewrite Equation (16) as:

(17) minimizeX,x Re(Tr∑k=1−NN−1ωkr−k(p)VkX)−λmax(R)Tr(X(p)X)=minimizex xH(C−λmax(R)x(p)(x(p))H)x

Here the matrix C has the form as follows:

(18) $$C=\sum_{k=1-N}^{N-1}\omega_{k}r_{-k}^{\left(p\right)}V_{k}=\sum_{k=1-N}^{0}\omega_{k}r_{-k}^{\left(p\right)}V_{k}+\sum_{k=1}^{N-1}\omega_{k}r_{-k}^{\left(p\right)}V_{k}=C_{1}+C_{2}$$

According to the definition in [22,23], C, $C_{1}$, and $C_{2}$ are all circulant matrices.

Through the above MM procedures, we decompose the quartic function of Equation (10) to the quadratic function of Equation (17). However, the quadratic function cannot get closed-form solution and needs to be further decomposed. As a result, we intend to use the MM strategy again and construct another majorization function to decompose the equation. In Lemma 1, both the object matrix and the surrogate matrix should be Hermitian matrix. To decompose Equation (17), we will introduce another lemma so that the surrogate function in Lemma 1 is also valid when the object matrix is a circulant matrix.  □

**Lemma** **2.** C *is the* N×N *circulant matrix,* $M_{2}$ *is the* N×N *Hermitian matrix, and* $M_{2}\geq C$ *. For each* $x_{0}$ *, the majorization function of* $x^{H}Cx$ *is similar with the conclusion in Lemma 1.*

With Lemma 2, the key to construct the majorization function of Equation (17) is to design an upperbound matrix $M_{2}$ so that $M_{2}\geq C-\lambda_{\max}\left(R\right)x^{\left(p\right)}{\left(x^{\left(p\right)}\right)}^{H}$. Similar with Equation (11), a simple way is to choose the upperbound matrix $M_{2}$ so that it can be expressed as:

(19) $$M_{2}=\lambda_{\max}\left(C-\lambda_{\max}\left(R\right)x^{\left(p\right)}{\left(x^{\left(p\right)}\right)}^{H}\right)I$$

Since $\lambda_{\max}\left(R\right)x^{\left(p\right)}{\left(x^{\left(p\right)}\right)}^{H}$ is a constant, we should focus on the eigenvalues of C. A column vector $c=C^{T}\left(1,:\right)$ is defined which is composed of the first row elements of matrix C. Also, the Fourier transform matrix is defined as:

(20) $$F=\left(f_{k,j}\right)=\exp\left(-i2\pi kj/N\right)\;0\leq k,j\leq N-1$$

Then all eigenvalues of C is solved as:

(21) $$\lambda \left(C\right)=F^{H}c$$

By Equations (20) and (21), the eigenvalues of $C,C_{1}$, and $C_{2}$ can be obtained by inverse Fourier transforms. Moreover, the circulant matrix ensures the relation between the maximum eigenvalues of $C,C_{1}$, and $C_{2}$ as [24]:

(22) $$\lambda_{\max}\left(C\right)=\lambda_{\max}\left(C_{1}+C_{2}\right)=\lambda_{\max}\left(C_{1}\right)+\lambda_{\max}\left(C_{2}\right)$$

Since $\lambda_{\max}\left(R\right)\geq 0$ in Equation (19), Equation (22) can be expanded as:

(23) $$\lambda_{\max}\left(C-\lambda_{\max}\left(R\right)x^{\left(p\right)}{\left(x^{\left(p\right)}\right)}^{H}\right)\leq \lambda_{\max}\left(C\right)=\lambda_{\max}\left(C_{1}\right)+\lambda_{\max}\left(C_{2}\right)$$

Now we define $\lambda_{c}=\lambda_{\max}\left(C_{1}\right)+\lambda_{\max}\left(C_{2}\right)$ and $M_{2}=\lambda_{c}I$ which is a Hermitian matrix. Since the matrix $\left(C-\lambda_{\max}\left(R\right)x^{\left(p\right)}{\left(x^{\left(p\right)}\right)}^{H}\right)$ is also circulant like C, we can now use Lemma 2 and the majorization function of Equation (17) can be yielded as:

(24) u2(x,x(p))=λcxHx+2Re(xH(C−λmax(R)x(p)(x(p))H−M2)x(p)) +(x(p))H(M2−C+λmax(R)x(p)(x(p))H)x(p)

Since $x^{H}x=N$, the first term of Equation (24) is a constant and the third term depends on the *p*th iteration only. Ignoring these constant terms, the original Equation can be surrogated by $u_{2}\left(x,x^{\left(p\right)}\right)$ as:

(25) minimizex Re(xH(C−λmax(R)x(p)(x(p))H−M2)x(p))

which can be simplified as follows:

(26) $$\underset{x}{\mathrm{minimize}}\;{\left\|x-y\right\|}_{2}$$

Here:

(27) $$\begin{array}{ll} y & =-\left(C-\lambda_{\max}\left(R\right)x^{\left(p\right)}{\left(x^{\left(p\right)}\right)}^{H}-M_{2}\right)x^{\left(p\right)}\\ & =\left(\lambda_{\max}\left(R\right)N+\lambda_{c}\right)x^{\left(p\right)}-Cx^{\left(p\right)} \end{array}$$

Equation (26) has a closed-form solution as:

(28) $$x=e^{j\arg\left(y\right)}$$

In order to improve the computation efficiency of MM procedures, we can express the WISL metric in Equation (3) and y in Equation (27) via the Fourier transform matrix. First of all, the periodic autocorrelation of a unimodular sequence can be written as follows [25]:

(29) $${\left[r_{0}^{\left(p\right)},r_{1}^{\left(p\right)},\ldots ,r_{N-1}^{\left(p\right)}\right]}^{T}=\frac{1}{N}F^{H}{\left|Fx^{\left(p\right)}\right|}^{2}$$

where the Fourier matrix F is defined as Equation (20). Then the WISL metric can be obtained through Equation (3). Furthermore, the computation of y depends on the matrix C in Equation (18) which can be expressed as follows [24]:

(30) $$C=\frac{1}{N}F^{H}\mathrm{Diag}\left(F^{H}c\right)F$$

where c is the column vector composed of the first row elements of C. 

The MM procedures of the method are presented as Table 1. During the procedures, it may not be definite to reach the steepest descent in each iteration, which results in a slow convergence speed. Hence, we modify the gradient direction in each iteration with the so called squared iterative method (SQUAREM) in order to improve the MM strategy and accelerate the convergence. The SQUAREM is derived from the Cauchy–Barzilai–Borwein algorithm [26] and originally used to accelerate the expectation–maximization (EM) algorithm in maximum likelihood estimates [27]. Recently, the SQUAREM is proved to be applicable for the MM strategy in aperiodic WISL case [17]. Since it is an ‘off-the-shelf’ acceleration scheme which needs nothing other than the parameter updating rules of MM procedures [16], we apply this scheme straightforward to accelerate our method. The details of the scheme can be found in [17]. Finally, the sequence design method based on MM procedures and the SQUAREM scheme is named as PeWISL method.

## 3. Simulations and Results

The performance of the proposed method is demonstrated via numerical simulations in this section. We prove the validity of the proposed method and compare its convergence with two relevant methods, including the PeCA method which also generates sequences with periodic WISL metric [19] and the method tackling the periodic ISL metric through the similar MM strategy and SQUAREM acceleration scheme (PeISL) [15]. Moreover, the matched filter performance of the PeWISL sequence is proposed and compared with the PeCA and the PeISL sequences. All of the simulations are conducted on a PC with a 3.50 GHz i7–3770K CPU and 4 GB RAM using MATLAB R2017a. The MATLAB code of PeCA is adopted from the monograph by He [8]. 

### 3.1. Designing Sequence with Low WISL Metric

For comparison, the sequence length and the weights of the WISL metric are in agreement with the set of the PeCA sequence in [19], which means a unimodular sequence with length N=512 will be generated and the weights are set as follows:

(31) $$\omega_{k}=\{\begin{array}{ll} 1, & \;k\in \left[1,0.12N\right]\\ 0, & \;\mathrm{otherwhise} \end{array}$$

**Figure 1:**
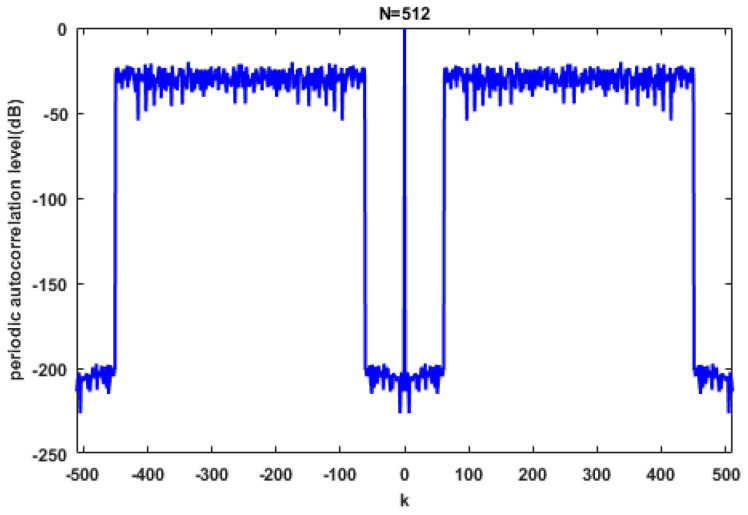
The autocorrelation levels of the unimodular sequence generated by the proposed PeWISL method. The sequence length is N=512 and the weights are set as Equation (31).

The initialization of the simulation is randomly generated and the stop criterion is set to be $\mathrm{WISL}\leq 10^{-10}$. Figure 1 shows the autocorrelation level of the PeWISL sequence. It is noticeable that the autocorrelation levels of weighted lags are lowered to −200 dB, which can be considered as almost zero. In addition, the correlations of PeWISL within the interested region do not go up when the time lag increases, which demonstrates that there is no existence of the ‘implicit weighting’ phenomenon in the PeCA sequence [19]. 

**Figure 2:**
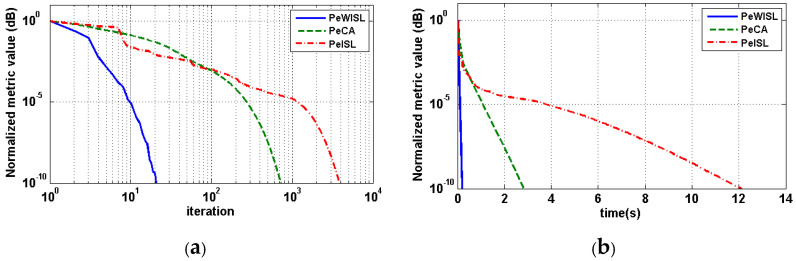
The evolution curves of the metric values with respect to (**a**) iterations and (**b**) consuming time of sequences with length N=128.

Figure 2 shows the evolution curves of three relevant methods in one simulation with respect to iterations and computation times. For comparison, all methods are initialized by the same randomly-generated sequence with length N=128. Furthermore, the normalized stopping criterions are set to $10^{-10}$. In Figure 2, it is obviously that the metric values of all three methods converge to $10^{-10}$. Both the WISL metric and the ISL metric can be well satisfied through this stopping criterion. It is worth noticing that the PeWISL consumes the least iterations which are just $10^{1}$ to reach the stopping criterion, followed by the PeCA with iterations more than $10^{2}$, and the PeISL with iterations more than $10^{3}$. This demonstrates the better computation efficiency of the PeWISL. Figure 2b illustrates the evolution curves of the metric values with respect to computation times. The result also shows that the PeWISL reaches the fastest consuming time of 0.20 s while the PeCA consumes 2.82 s. On the contrary, the PeISL method with the same acceleration scheme reaches the largest computation times of 12.08 s, which shows the difficulty to suppress autocorrelation sidelobes of all the time lags with the periodic ISL metric. This explains why we focus on the optimization of the periodic WISL metric. Also, the huge difference between the computation times of the PeWISL and the PeISL testifies the larger acceleration efficiency of the SQUAREM on the PeWISL. In addition, we notice that there are some unsmooth distortions in the evolution curves of the PeWISL and the PeISL. The reasons are as follows: (1) Due to the nature of the MM strategy, the decent gradients of the PeWISL and the PeISL are nonuniform compared with PeCA based on the cyclic algorithm. (2) Since the PeWISL consumes only $10^{1}$ iterations to reduce the metric values by $10^{10}$, its sampling points are not enough to fit a smooth curve.

Table 2 lists the iterations and computation times of three methods to generate sequences with three different lengths. All the results in the table are the average values of 100 simulations. The weights of the PeWISL and the PeCA sequences are set as Equation (31). As the sequence length increases, the iterations and computation times of methods increase significantly, except for the PeWISL. It can be seen that the PeWISL consumes the least computation time and the fewest iterations in all three lengths, even compared with the PeCA satisfying the same periodic WISL metric and the PeISL which is also based on the SQUAREM acceleration scheme. Combined Table 2 with Figure 2, we can see that the iteration of PeWISL floats just from 20 to 37 when the length increases from 128 (Figure 2) to 1024. On the contrary, the PeCA and the PeISL consume more iterations which are nearly proportional to the length when N≤512. Particularly, the PeISL with N=1024 consumes iterations and computation time more than $10^{6}$. In brief, the relatively stable iteration and less computation time of PeWISL reveal its higher computational efficiency than other methods. 

### 3.2. Matched Filter Performance

Recently, most active sonar systems utilize the matched filter to estimate the range and velocity of targets through the correlation between replicas and echoes. The matched filter provides the highest signal-noise-ratio in the white noised environment. In addition, it is computationally efficient and simple because of the FFT operations. In this section, we propose several simulations to evaluate the matched filter performance of the PeWISL phase-coded sequences, especially the matched filter results versus ranges. The comparison with the PeCA and the PeISL (PeCAN) sequence is also demonstrated through simulations.

**Figure 3:**
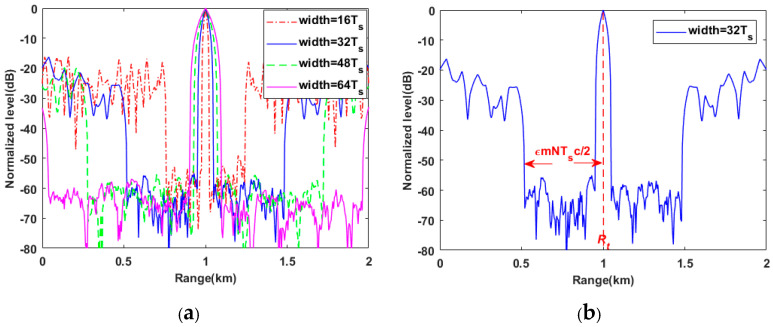
(**a**) The matched filter results versus ranges for the PeWISL sequences with coding width $16T_{s},32T_{s},48T_{s},64T_{s}$; (**b**) the optimized range interval corresponding with range interval (32) when the coding width is $32T_{s}$.

**Simulation 1:** The weights of the WISL metric in this simulation are set as [1,0.1N]. The carrier frequency is $f_{0}=500\mathrm{Hz}$ and the sequence length is fixed to N=100. The stationary target is located 1 km away from the receiver. In this simulation, the coding width of the sequence change by $16T_{s},32T_{s},48T_{s}$, and $64T_{s}$ respectively, where $T_{s}=1/f_{0}$ represents the carrier period. Figure 3a presents the matched filter results versus ranges with different coding widths. With the increasing of coding widths $mT_{s}$ (m denotes a positive integer), the total durations of sequences ($T=N\times mT_{s}$) increase from 3.2 s (coding width $16T_{s}$) to 12.8 s (coding width $64T_{s}$). As a result, the length of the optimized range interval also increases, which means the improvement of the ability to detect weak targets in a given range interval. Meanwhile, the increasing of the mainlobe width in Figure 3a means that the range resolution is lowered and results in the difficulty of distinguishing other targets near the main target. This suggests a trade-off between detecting weak targets and distinguishing multi-targets. Theoretically, when the weights are set as [1,εN],0<ε<1, the optimized range interval of a phase-coded sequence with length N, carrier frequency $f_{0}$ and coding width $mT_{s}$ can be represented as follows:

(32) $$\left[R_{t}-\frac{\varepsilon mNT_{s}}{2}c,R_{t}+\frac{\varepsilon mNT_{s}}{2}c\right]$$

where $R_{t}$ is the distance between the target and the receiver, c denotes the underwater sound velocity. Figure 3b shows the length of the optimized range interval when coding width is $32T_{s}$. It is clearly that the optimized range interval is [0.52,1.48] km, which is corresponding with the quantitative expression of range interval (32).

**Figure 4:**
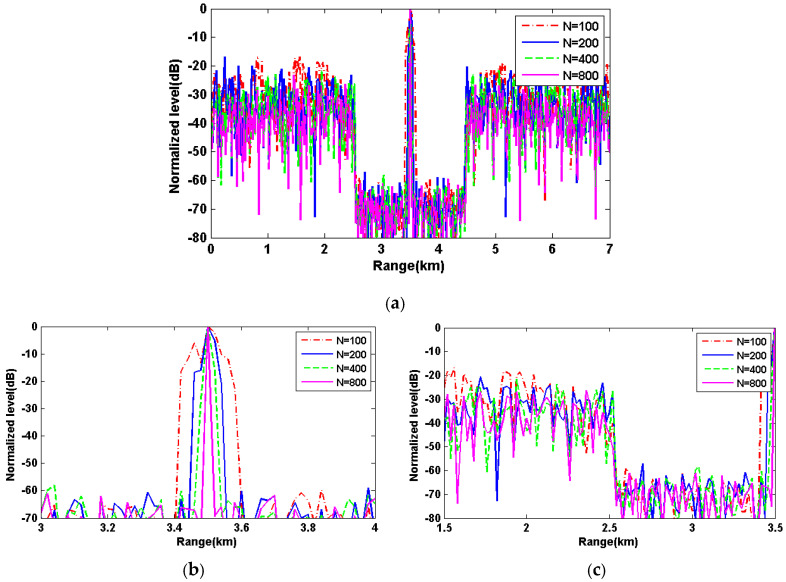
(**a**) The matched filter results versus ranges for the PeWISL sequences with sequence length 100, 200, 400, and 800, respectively; (**b**) the details of mainlobes with different sequence lengths; and (**c**) the details of sidelobes in the range interval [1.5,3.5] km.

**Simulation 2:** The carrier frequency is $f_{0}=500\mathrm{Hz}$ and the sequence duration is fixed to 12.8 s. The weights are the same as simulation 1. The stationary target is located 3.5 km away from the receiver. In this simulation, the sequence length change by N=100,200,400, and 800, respectively. Figure 4 shows the matched filter results versus ranges with different sequence lengths. In Figure 4a, it can be seen that the optimized range interval remains unaltered since the total duration is unchanged. Figure 4b shows that the width of mainlobe decreases as length N increases, which results in the enhanced range resolution. Figure 4c shows the details of sidelobes in the range intervals [1.5,3.5] km. As length N increases, the average sidelobe levels in the unoptimized range intervals get lowered. 

In the practical application of matched filters, the optimized range interval of PeWISL is expected to contain the probable target location so that the precise target location can be extracted. In particular, if the interval of the probable target location is $\left[l_{1},l_{2}\right]$ via the prior information, we define $R_{t}=\frac{l_{1}+l_{2}}{2}$ as the ‘standard position’. Then the interval $\left[l_{1},l_{2}\right]$ can be rewritten as $[R_{t}-\frac{l_{2}-l_{1}}{2},R_{t}+\frac{l_{2}-l_{1}}{2}]$. The optimized range interval should contain the probable target location which means the following inequality could be satisfied:

(33) $$\frac{l_{2}-l_{1}}{2}\leq \frac{\varepsilon mNT_{s}}{2}c$$

Then ε in [1,εN] should set as:

(34) $$\frac{l_{2}-l_{1}}{mcNT_{s}}\leq \varepsilon <1$$

By this way, the weights of the PeWISL [1,εN] can set in real time according to the priori information. Then the optimized range interval is guaranteed to contain the target and the PeWISL transmitted sequence can get the target range precisely.

**Simulation 3:** In this simulation, we compare the matched filter performances of the PeWISL sequence with other two phased-coded sequences, the PeCA sequence and the PeISL (PeCAN) sequence. Since the PeISL and the PeCAN sequences both focus on the periodic ISL metric and reach the similar optimization performance [14,17], we consider them as one representation in this paper. All sequences have a carrier frequency $f_{0}=500\mathrm{Hz}$, a sequence length of N=100, a sequence duration T=6.4s and a coding width $64T_{s}$. The set of weights for the PeWISL and PeCA are [1,0.075N]. The simulated target was 2 km away from the receiver with a radial velocity of 9 km/h. According to the result of range interval (32), the optimized range interval is [1.28,2.72] km. In order to satisfy the periodic condition, both sequences are transmitted continuously and periodically with three duty cycles. 

**Figure 5:**
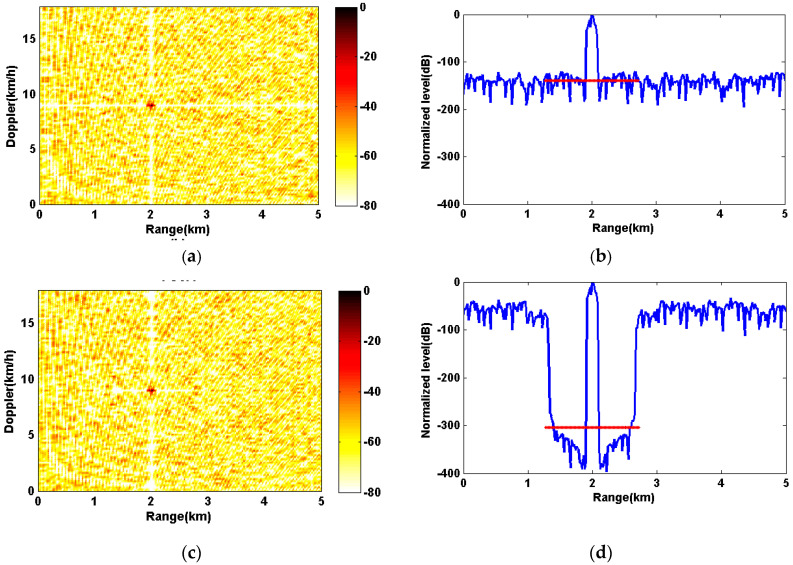

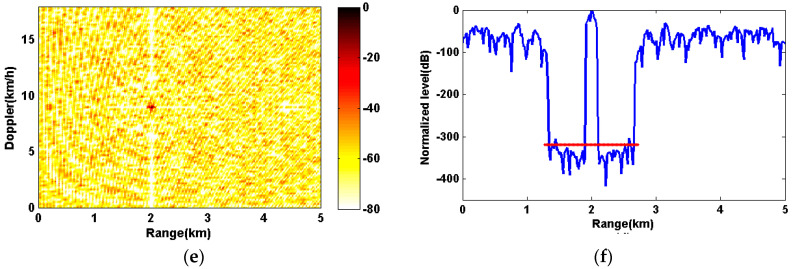
The matched filter performance of the PeISL (PeCAN), PeCA, and the PeWISL sequences. Each sequence has the same carrier frequency, sequence length, and coding width. (**a**,**c**,**e**) are the range-Doppler imaging results of three sequences respectively. (**b**,**d**,**f**) are the match filter results versus range. The horizontal solid red lines represent the average sidelobe level in the optimized range interval [1.28,2.72] km.

Figure 5 shows the matched filter performance of three sequences. Figure 5a,c,e are the range-Doppler imaging results. All three sequences reach the high resolution in range and Doppler. For the PeISL (PeCAN) of Figure 5a, there are two low sidelobe intervals across the true target position. By contrast, the PeCA in Figure 5c and the PeWISL in Figure 5e show the short interval of low sidelobes and the same sharpness of the target ‘lightspot’. Since the PeCA and the PeWISL only focus on the reduction of sidelobes in time domain, it is reasonable that there is no difference between the match filter results versus Doppler of three methods. For simplicity, we only present the match filter results versus range in Figure 5b,d,f. In Figure 5f, the average sidelobe level of PeWISL in the whole range interval is −130.09 dB which is between −143.47 dB of PeISL (PeCAN) in Figure 5b and −118.26 dB of PeCA in Figure 5d. On the contrary, the average sidelobe level of the PeWISL in the optimized range interval is suppressed to −318.01 dB (the solid red line in Figure 5f), which is the lowest compared with the PeISL (PeCAN) (−132.90 dB) and the PeCA (−304.77 dB). The results indicate that the PeWISL sequence sacrifices the average ISL in the whole range interval for the lowest sidelobe levels in the optimized interval. This also demonstrates that among the methods optimizing the periodic WISL metric, the PeWISL reaches the better matched filter performance than the PeCA.

## 4. Conclusions

In this paper, a phase-coded sequence design method namely PeWISL is presented which is based on the MM strategy. For the first time, we affirm that the MM strategy can be applied to the sequence design method of periodic WISL metric. Through simulations, the validity of the method is proved and the convergence properties are compared with two relevant methods. The comparison results reveal that the PeWISL method promotes the convergence efficiency and decreases the computation time and iterations greatly. In addition, the matched filter performances of the generated sequences are also evaluated. In the practical matched filter processing, the PeWISL method can suppress sidelobe levels of the specific range interval to considerably low, which is the sacrifice of sidelobe levels in uninterested range intervals. Overall, the proposed method improves the efficiency and the real-time capacity of the phased-coded sequence design, which makes the PeWISL sequence an applicable periodic transmitted sequence for active sonars.

We identify three potential avenues for future works. The first is to conduct the quantitative analyses on convergence of the methods and explore factors which are highly correlated with the convergence performance. For example, the distance between upperbound matrices and the objective matrix. The second is to expand the application scope of the method into the sequence design of MIMO sonar systems. The third is to verify the performance of the PeWISL sequence through sea-trial experiments.

## Figures and Tables

**Table 1:** Majorization–minimization (MM) procedures of the periodic Weighted Integrated Sidelobe Level (PeWISL) method.

**Initials:**p=0, sequence length N, initial $x^{\left(0\right)}$, weights $\left\{\omega_{k}\geq 0|k=1,\ldots ,N-1\right\}$, $\lambda_{\max}\left(R\right)=\underset{k}{\max}\left\{N\left(\omega_{k}+\omega_{N-k}\right)|k=1,\ldots ,N-1\right\}$
**Repeat**
1: $f=Fx^{\left(p\right)}$
2: $r=\frac{1}{N}F^{H}{\left|f\right|}^{2}$
3: $c_{1}=\left[r_{0},r_{-1},\ldots ,r_{1-N}\right]\circ {\left[0,\omega_{1},\ldots ,\omega_{N-1}\right]}^{T}$ $c_{2}=\left[r_{0},r_{N-1},\ldots ,r_{1}\right]\circ {\left[0,\omega_{N-1},\ldots ,\omega_{1}\right]}^{T}$
4: $c=c_{1}+c_{2}$
5: $\lambda \left(C_{1}\right)=F^{*}c_{1}$,$\lambda \left(C_{2}\right)=F^{*}c_{2}$
6: $\lambda_{c}=\lambda_{\max}\left(C_{1}\right)+\lambda_{\max}\left(C_{2}\right)$
7: $y=\left(\left(\lambda_{\max}\left(R\right)N+\lambda_{c}\right)-\frac{1}{N}F^{H}\mathrm{Diag}\left(F^{H}c\right)F\right)x^{\left(p\right)}$
8: $x_{n}^{\left(p+1\right)}=e^{j\arg\left(y_{n}\right)},n=1,\ldots ,N$
9: p=p+1
**Until** convergence

**Table 2:** The convergence performance of three methods.

	N=256		N=512		N=1024	
	Iteration	Time (s)	Iteration	Time (s)	Iteration	Time (s)
PeWISL	33	0.88	31	3.84	37	34.98
PeCA	2611	8.28	4538	45.76	8375	333.66
PeISL	20641	61.54	45821	969.52	$∼10^{6}$	$∼10^{6}$

Iteration: convergence iterations. Time: computation time (in seconds).
